# Diiodido(1,10-phenanthroline-5,6-dione-κ^2^ *N*,*N*′)mercury(II)

**DOI:** 10.1107/S1600536811038748

**Published:** 2011-09-30

**Authors:** Akbar Ghaemi, Rezvan Shojaiean, Seik Weng Ng, Edward R. T. Tiekink

**Affiliations:** a Department of Chemistry, Saveh Branch, Islamic Azad University, Saveh, Iran; b Department of Chemistry, University of Malaya, 50603 Kuala Lumpur, Malaysia; c Chemistry Department, Faculty of, Science, King Abdulaziz University, PO Box 80203 Jeddah, Saudi Arabia

## Abstract

The Hg^II^ atom in the title complex, [HgI_2_(C_12_H_6_N_2_O_2_)], is tetra­hedrally coordinated by the N atoms of the chelating 1,10-phenanthroline-5,6-dione ligand and two I atoms. The range of tetra­hedral angles is broad, *viz*. 68.94 (17)° for the chelate angle to a wide 132.627 (15)° for the I—Hg—I angle. The ligand mol­ecule is non-planar with the O atoms lying 0.422 (5) and −0.325 (5) Å out of the plane through the remaining atoms [r.m.s. deviation = 0.068 Å]. Mol­ecules are consolidated in the crystal packing by C—H⋯O inter­actions.

## Related literature

For the ligand synthesis and the crystal structure of 1,10-phenanthroline-5,6-dione, see: Calderazzo *et al.* (1999 ▶). For an evaluation of the different coordinating ability of the two sets of donor atoms in the ligand, see: Fujihara *et al.* (2003 ▶). For the structure of the dichlorido analogue with two 1,10-phenanthroline-5,6-dione ligands, see: Figueiras *et al.* (2009 ▶). For the crystallization procedure, see: Harrowfield *et al.* (1996 ▶).
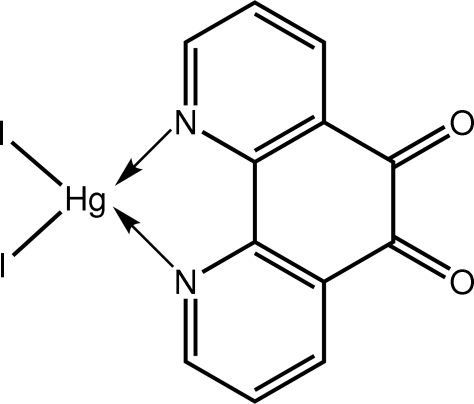


## Experimental

### 

#### Crystal data

[HgI_2_(C_12_H_6_N_2_O_2_)]
*M* *_r_* = 664.58
Monoclinic, 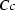

*a* = 11.7941 (3) Å
*b* = 8.1725 (1) Å
*c* = 15.3982 (3) Å
β = 108.298 (2)°
*V* = 1409.14 (5) Å^3^
*Z* = 4
Mo *K*α radiation
μ = 15.30 mm^−1^
*T* = 100 K
0.15 × 0.15 × 0.15 mm

#### Data collection

Agilent SuperNova Dual diffractometer with Atlas detector
Absorption correction: multi-scan (*CrysAlis PRO*; Agilent, 2010 ▶) *T*_min_ = 0.586, *T*_max_ = 1.000
15631 measured reflections
3209 independent reflections
3155 reflections with *I* > 2σ(*I*)
*R*_int_ = 0.041

#### Refinement

*R*[*F* ^2^ > 2σ(*F* ^2^)] = 0.022
*wR*(*F* ^2^) = 0.049
*S* = 1.03
3209 reflections
172 parameters
2 restraints
H-atom parameters constrained
Δρ_max_ = 0.51 e Å^−3^
Δρ_min_ = −0.79 e Å^−3^
Absolute structure: Flack (1983 ▶), 1579 Friedel pairs
Flack parameter: −0.005 (3)

### 

Data collection: *CrysAlis PRO* (Agilent, 2010 ▶); cell refinement: *CrysAlis PRO*; data reduction: *CrysAlis PRO*; program(s) used to solve structure: *SHELXS97* (Sheldrick, 2008 ▶); program(s) used to refine structure: *SHELXL97* (Sheldrick, 2008 ▶); molecular graphics: *ORTEP-3* (Farrugia, 1997 ▶) and *DIAMOND* (Brandenburg, 2006 ▶); software used to prepare material for publication: *publCIF* (Westrip, 2010 ▶).

## Supplementary Material

Crystal structure: contains datablock(s) general, I. DOI: 10.1107/S1600536811038748/hg5098sup1.cif

Structure factors: contains datablock(s) I. DOI: 10.1107/S1600536811038748/hg5098Isup2.hkl

Additional supplementary materials:  crystallographic information; 3D view; checkCIF report

## Figures and Tables

**Table 1:** Selected bond lengths (Å)

Hg1—N1	2.411 (5)
Hg1—N2	2.416 (5)
Hg1—I1	2.6637 (4)
Hg1—I2	2.6739 (4)

**Table 2:** Hydrogen-bond geometry (Å, °)

*D*—H⋯*A*	*D*—H	H⋯*A*	*D*⋯*A*	*D*—H⋯*A*
C1—H1⋯O2^i^	0.95	2.58	3.077 (8)	113
C12—H12⋯O1^ii^	0.95	2.40	3.248 (7)	148

Symmetry codes: (i) 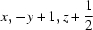; (ii) 
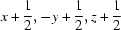
.

**Fig. 1.**
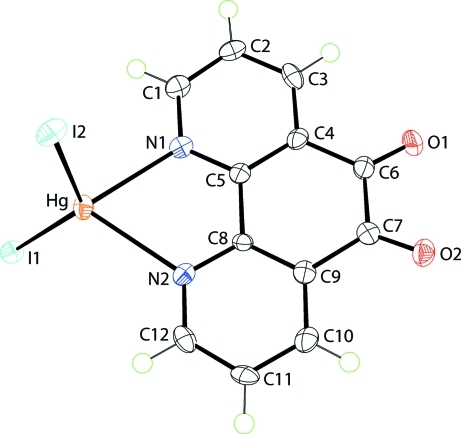
The molecular structure of (I) showing the atom-labelling scheme and displacement ellipsoids at the 70% probability level.

**Fig. 2.**
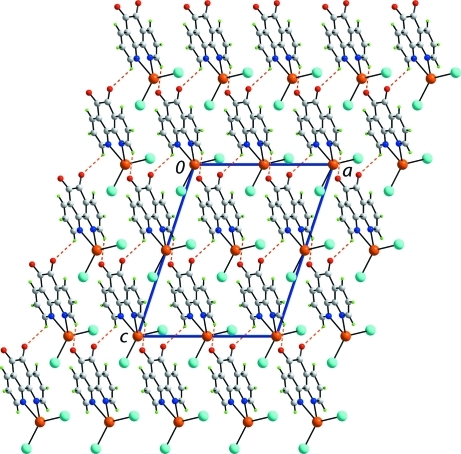
A view in projection down the b axis of the unit-cell contents of (I). The C—H···O interactions are shown as orange dashed lines.

## supplementary crystallographic information

### Comment

1,10-Phenanthroline-5,6-dione (Calderazzo *et al.*, 1999) has attracted our attention due to the presence of two coordinating functionalities within the same molecule, *i.e*. the quinonoid and the diimine residues. Moreover, the presence of two types of basic centres, *i.e*. nitrogen and oxygen, both *sp^2^*-hybridized, makes this molecule an ideal system to study the different coordinating ability of the two sets of donor atoms (Calderazzo *et al.*, 1999; Fujihara *et al.*, 2003). In connection with a recent structure determination of the related mercury(II)dichlorido structure with two 1,10-phenanthroline-5,6-dione ligands (Figueiras *et al.*, 2009), the structure of the title compound, (I), was determined.

The Hg atom in (I), Fig. 1 and Table 1, is chelated by the 1,10-phenanthroline-5,6-dione ligand and the distorted tetrahedral I_2_N_2_ donor set is completed by two I atoms. The range of tetrahedral angles is from a narrow 68.94 (17)°, for the chelate angle, to a wide 132.627 (15)°, for the angle subtended at Hg by the I atoms. The 1,10-phenanthroline-5,6-dione ligand is planar with the r.m.s. deviation for the 14 C and N atoms being 0.068 Å with the maximum deviations from the least-squares plane being 0.146 (6) Å for atom C7 and -0.116 (7) for atom C6; the O1 and O2 atoms lie -0.325 (5) and 0.422 (5) Å out of this plane, respectively.

The molecules of (I) are consolidated in the crystal packing *via* weak C—H···O interactions involving both O atoms, Table 2 and Fig. 2.

### Experimental

The title complex was obtained by the branched tube method (Harrowfield *et al.*, 1996). 1,10-Phenanthroline-5,6-dione (0.136 g, 0.648 mmol) and HgI_2_ (0.294 g, 0648 mmol) were placed at the bottom of main arm of a branched tube. Methanol was carefully added to fill both arms. The tube was sealed and the main arm immersed in a bath at 333 K while the other was kept at ambient temperature. After five days, red crystals were deposited in the cooler arm. These were filtered off, washed with acetone and ether, and air dried; Yield: 60%; *M*.pt. 524–526 K. Anal. Calc for C_12_H_6_HgI_2_N_2_O_2_; C, 21.7, H, 0.60, N, 4.2%; Found: C, 21.89, H, 0.69, N: 4.24%. Selected FT—IR data, ν(cm^-1^): 1687 (C═O). ^1^H NMR (δ): 7.85–7.89 (dd, 2H, H3,8), 8.53–8.56 (dd, 2H, H2,9), 9.01–9.03 (dd, 2H, H4,7) p.p.m..

### Refinement

The H-atoms were placed in calculated positions (C—H 0.95 Å) and were included in the refinement in the riding model approximation, with *U*_iso_(H) set to 1.2*U*_equiv_(C). A number of reflections, *i.e*. 4 0 -10, 1 3 -10, 6 0 -10, 0 0 8, 2 4 -11, 6 0 8, 5 3 -14 and 3 3 -12, were omitted from the final refinement owing to poor agreement.

### Crystal data

**Table:**

[HgI_2_(C_12_H_6_N_2_O_2_)]	*F*(000) = 1176
*M**_r_* = 664.58	*D*_x_ = 3.133 Mg m^−^^3^
Monoclinic, *C**c*	Mo *K*α radiation, λ = 0.71073 Å
Hall symbol: C -2yc	Cell parameters from 11298 reflections
*a* = 11.7941 (3) Å	θ = 2.8–29.3°
*b* = 8.1725 (1) Å	µ = 15.30 mm^−^^1^
*c* = 15.3982 (3) Å	*T* = 100 K
β = 108.298 (2)°	Block, red
*V* = 1409.14 (5) Å^3^	0.15 × 0.15 × 0.15 mm
*Z* = 4	

### Data collection

**Table:**

Agilent SuperNova Dual diffractometer with Atlas detector	3209 independent reflections
Radiation source: SuperNova (Mo) X-ray Source	3155 reflections with *I* > 2σ(*I*)
Mirror	*R*_int_ = 0.041
Detector resolution: 10.4041 pixels mm^-1^	θ_max_ = 27.5°, θ_min_ = 2.8°
ω scan	*h* = −15→15
Absorption correction: multi-scan (*CrysAlis PRO*; Agilent, 2010)	*k* = −10→10
*T*_min_ = 0.586, *T*_max_ = 1.000	*l* = −19→19
15631 measured reflections	

### Refinement

**Table:**

Refinement on *F*^2^	Secondary atom site location: difference Fourier map
Least-squares matrix: full	Hydrogen site location: inferred from neighbouring sites
*R*[*F*^2^ > 2σ(*F*^2^)] = 0.022	H-atom parameters constrained
*wR*(*F*^2^) = 0.049	*w* = 1/[σ^2^(*F*_o_^2^) + (0.0292*P*)^2^] where *P* = (*F*_o_^2^ + 2*F*_c_^2^)/3
*S* = 1.03	(Δ/σ)_max_ = 0.001
3209 reflections	Δρ_max_ = 0.51 e Å^−^^3^
172 parameters	Δρ_min_ = −0.79 e Å^−^^3^
2 restraints	Absolute structure: Flack (1983), 1579 Friedel pairs
Primary atom site location: structure-invariant direct methods	Flack parameter: −0.005 (3)

### Special details

**Table:**

Geometry. All e.s.d.'s (except the e.s.d. in the dihedral angle between two l.s. planes) are estimated using the full covariance matrix. The cell e.s.d.'s are taken into account individually in the estimation of e.s.d.'s in distances, angles and torsion angles; correlations between e.s.d.'s in cell parameters are only used when they are defined by crystal symmetry. An approximate (isotropic) treatment of cell e.s.d.'s is used for estimating e.s.d.'s involving l.s. planes.
Refinement. Refinement of *F*^2^ against ALL reflections. The weighted *R*-factor *wR* and goodness of fit *S* are based on *F*^2^, conventional *R*-factors *R* are based on *F*, with *F* set to zero for negative *F*^2^. The threshold expression of *F*^2^ > σ(*F*^2^) is used only for calculating *R*-factors(gt) *etc*. and is not relevant to the choice of reflections for refinement. *R*-factors based on *F*^2^ are statistically about twice as large as those based on *F*, and *R*- factors based on ALL data will be even larger.

### Fractional atomic coordinates and isotropic or equivalent isotropic displacement parameters (Å^2^)

**Table:**

	*x*	*y*	*z*	*U*_iso_*/*U*_eq_	
Hg1	0.499993 (19)	0.48034 (2)	0.500006 (16)	0.01421 (6)	
I1	0.46538 (3)	0.38365 (4)	0.65496 (2)	0.01434 (9)	
I2	0.66399 (3)	0.67286 (5)	0.46534 (3)	0.01911 (10)	
O1	0.0663 (4)	0.5129 (5)	0.0820 (3)	0.0211 (10)	
O2	0.2083 (4)	0.2572 (5)	0.0607 (3)	0.0174 (9)	
N1	0.3288 (4)	0.5884 (6)	0.3831 (3)	0.0132 (10)	
N2	0.4235 (4)	0.2848 (6)	0.3779 (3)	0.0120 (10)	
C1	0.2862 (5)	0.7383 (7)	0.3878 (4)	0.0154 (12)	
H1	0.3175	0.7988	0.4428	0.018*	
C2	0.1973 (6)	0.8101 (7)	0.3152 (4)	0.0160 (12)	
H2	0.1679	0.9164	0.3208	0.019*	
C3	0.1537 (5)	0.7221 (8)	0.2351 (4)	0.0156 (12)	
H3	0.0938	0.7673	0.1842	0.019*	
C4	0.1990 (6)	0.5657 (7)	0.2299 (4)	0.0132 (11)	
C5	0.2882 (6)	0.5030 (6)	0.3057 (4)	0.0111 (11)	
C6	0.1530 (6)	0.4715 (7)	0.1439 (4)	0.0142 (12)	
C7	0.2238 (5)	0.3175 (7)	0.1356 (4)	0.0115 (11)	
C8	0.3413 (5)	0.3392 (7)	0.3019 (4)	0.0100 (11)	
C9	0.3084 (5)	0.2485 (7)	0.2209 (4)	0.0130 (12)	
C10	0.3582 (5)	0.0953 (7)	0.2196 (4)	0.0156 (12)	
H10	0.3378	0.0324	0.1650	0.019*	
C11	0.4383 (6)	0.0363 (7)	0.2996 (4)	0.0147 (12)	
H11	0.4713	−0.0702	0.3019	0.018*	
C12	0.4696 (6)	0.1365 (8)	0.3764 (4)	0.0155 (12)	
H12	0.5267	0.0971	0.4307	0.019*	

### Atomic displacement parameters (Å^2^)

**Table:**

	*U*^11^	*U*^22^	*U*^33^	*U*^12^	*U*^13^	*U*^23^
Hg1	0.01526 (11)	0.01650 (11)	0.01011 (10)	−0.00067 (10)	0.00290 (7)	−0.00105 (10)
I1	0.01624 (19)	0.01475 (19)	0.01394 (18)	−0.00041 (15)	0.00747 (15)	−0.00073 (14)
I2	0.0206 (2)	0.01604 (19)	0.0225 (2)	−0.00531 (16)	0.00937 (17)	−0.00433 (16)
O1	0.023 (3)	0.019 (2)	0.015 (2)	−0.0015 (18)	−0.0049 (19)	0.0022 (17)
O2	0.021 (2)	0.018 (2)	0.012 (2)	−0.0045 (18)	0.0053 (17)	−0.0010 (18)
N1	0.012 (2)	0.013 (2)	0.015 (2)	−0.001 (2)	0.007 (2)	−0.001 (2)
N2	0.014 (2)	0.012 (2)	0.012 (2)	−0.0029 (19)	0.0064 (19)	−0.0014 (19)
C1	0.017 (3)	0.015 (3)	0.016 (3)	0.000 (2)	0.009 (3)	−0.002 (2)
C2	0.017 (3)	0.011 (3)	0.021 (3)	−0.003 (2)	0.009 (3)	−0.002 (2)
C3	0.013 (3)	0.021 (3)	0.012 (3)	0.000 (2)	0.004 (2)	0.006 (2)
C4	0.015 (3)	0.012 (3)	0.013 (3)	−0.005 (2)	0.005 (2)	−0.001 (2)
C5	0.013 (3)	0.011 (3)	0.011 (3)	−0.004 (2)	0.006 (2)	0.001 (2)
C6	0.018 (3)	0.014 (3)	0.011 (3)	−0.005 (2)	0.005 (3)	0.001 (2)
C7	0.009 (3)	0.013 (3)	0.014 (3)	−0.004 (2)	0.005 (2)	0.002 (2)
C8	0.010 (3)	0.008 (2)	0.012 (3)	0.000 (2)	0.003 (2)	0.001 (2)
C9	0.015 (3)	0.013 (3)	0.012 (3)	−0.005 (2)	0.006 (2)	0.002 (2)
C10	0.018 (3)	0.014 (3)	0.015 (3)	−0.007 (2)	0.005 (2)	−0.001 (2)
C11	0.014 (3)	0.009 (3)	0.021 (3)	−0.001 (2)	0.006 (3)	0.005 (2)
C12	0.014 (3)	0.019 (3)	0.012 (3)	−0.001 (2)	0.004 (2)	0.006 (2)

### Geometric parameters (Å, °)

**Table:**

Hg1—N1	2.411 (5)	C3—C4	1.398 (9)
Hg1—N2	2.416 (5)	C3—H3	0.9500
Hg1—I1	2.6637 (4)	C4—C5	1.400 (9)
Hg1—I2	2.6739 (4)	C4—C6	1.479 (8)
O1—C6	1.207 (8)	C5—C8	1.487 (8)
O2—C7	1.215 (7)	C6—C7	1.538 (8)
N1—C5	1.333 (8)	C7—C9	1.488 (8)
N1—C1	1.335 (8)	C8—C9	1.396 (8)
N2—C8	1.341 (7)	C9—C10	1.386 (9)
N2—C12	1.331 (8)	C10—C11	1.382 (9)
C1—C2	1.399 (9)	C10—H10	0.9500
C1—H1	0.9500	C11—C12	1.390 (9)
C2—C3	1.380 (9)	C11—H11	0.9500
C2—H2	0.9500	C12—H12	0.9500
			
N2—Hg1—N1	68.94 (17)	N1—C5—C4	121.3 (5)
N2—Hg1—I1	112.07 (11)	N1—C5—C8	118.0 (5)
N1—Hg1—I1	116.72 (11)	C4—C5—C8	120.7 (5)
N2—Hg1—I2	110.55 (11)	O1—C6—C4	122.9 (6)
N1—Hg1—I2	97.13 (12)	O1—C6—C7	120.7 (5)
I1—Hg1—I2	132.627 (15)	C4—C6—C7	116.4 (5)
C5—N1—C1	119.5 (5)	O2—C7—C9	123.0 (5)
C5—N1—Hg1	117.2 (4)	O2—C7—C6	119.2 (5)
C1—N1—Hg1	122.5 (4)	C9—C7—C6	117.9 (5)
C8—N2—C12	118.3 (5)	N2—C8—C9	121.7 (5)
C8—N2—Hg1	116.8 (4)	N2—C8—C5	117.5 (5)
C12—N2—Hg1	124.1 (4)	C9—C8—C5	120.7 (5)
N1—C1—C2	122.8 (6)	C10—C9—C8	119.5 (6)
N1—C1—H1	118.6	C10—C9—C7	120.0 (5)
C2—C1—H1	118.6	C8—C9—C7	120.5 (5)
C3—C2—C1	118.1 (6)	C9—C10—C11	118.5 (6)
C3—C2—H2	120.9	C9—C10—H10	120.8
C1—C2—H2	120.9	C11—C10—H10	120.8
C2—C3—C4	119.1 (6)	C10—C11—C12	118.5 (6)
C2—C3—H3	120.5	C10—C11—H11	120.8
C4—C3—H3	120.5	C12—C11—H11	120.8
C5—C4—C3	119.1 (6)	N2—C12—C11	123.4 (6)
C5—C4—C6	121.6 (5)	N2—C12—H12	118.3
C3—C4—C6	119.2 (6)	C11—C12—H12	118.3
			
N2—Hg1—N1—C5	−9.4 (4)	C5—C4—C6—C7	−11.3 (8)
I1—Hg1—N1—C5	−114.3 (4)	C3—C4—C6—C7	167.7 (5)
I2—Hg1—N1—C5	99.9 (4)	O1—C6—C7—O2	15.4 (8)
N2—Hg1—N1—C1	−179.3 (5)	C4—C6—C7—O2	−163.1 (5)
I1—Hg1—N1—C1	75.8 (5)	O1—C6—C7—C9	−164.2 (6)
I2—Hg1—N1—C1	−69.9 (4)	C4—C6—C7—C9	17.3 (7)
N1—Hg1—N2—C8	10.8 (4)	C12—N2—C8—C9	−3.4 (8)
I1—Hg1—N2—C8	122.1 (4)	Hg1—N2—C8—C9	167.0 (4)
I2—Hg1—N2—C8	−79.1 (4)	C12—N2—C8—C5	178.2 (5)
N1—Hg1—N2—C12	−179.4 (5)	Hg1—N2—C8—C5	−11.3 (6)
I1—Hg1—N2—C12	−68.1 (5)	N1—C5—C8—N2	2.7 (8)
I2—Hg1—N2—C12	90.8 (5)	C4—C5—C8—N2	−177.9 (5)
C5—N1—C1—C2	1.7 (9)	N1—C5—C8—C9	−175.7 (5)
Hg1—N1—C1—C2	171.3 (4)	C4—C5—C8—C9	3.8 (8)
N1—C1—C2—C3	−0.9 (9)	N2—C8—C9—C10	2.5 (8)
C1—C2—C3—C4	0.4 (9)	C5—C8—C9—C10	−179.2 (5)
C2—C3—C4—C5	−0.6 (9)	N2—C8—C9—C7	−175.3 (5)
C2—C3—C4—C6	−179.6 (5)	C5—C8—C9—C7	3.0 (8)
C1—N1—C5—C4	−1.8 (8)	O2—C7—C9—C10	−10.9 (8)
Hg1—N1—C5—C4	−172.0 (4)	C6—C7—C9—C10	168.7 (5)
C1—N1—C5—C8	177.6 (5)	O2—C7—C9—C8	166.9 (5)
Hg1—N1—C5—C8	7.4 (6)	C6—C7—C9—C8	−13.6 (8)
C3—C4—C5—N1	1.3 (9)	C8—C9—C10—C11	0.9 (9)
C6—C4—C5—N1	−179.7 (5)	C7—C9—C10—C11	178.6 (5)
C3—C4—C5—C8	−178.1 (5)	C9—C10—C11—C12	−3.0 (9)
C6—C4—C5—C8	0.8 (9)	C8—N2—C12—C11	1.1 (9)
C5—C4—C6—O1	170.3 (6)	Hg1—N2—C12—C11	−168.6 (4)
C3—C4—C6—O1	−10.8 (9)	C10—C11—C12—N2	2.1 (9)

### Hydrogen-bond geometry (Å, °)

**Table:**

*D*—H···*A*	*D*—H	H···*A*	*D*···*A*	*D*—H···*A*
C1—H1···O2^i^	0.95	2.58	3.077 (8)	113
C12—H12···O1^ii^	0.95	2.40	3.248 (7)	148

Symmetry codes: (i) *x*, −*y*+1, *z*+1/2; (ii) *x*+1/2, −*y*+1/2, *z*+1/2.
